# Extra Supplement.—The Nursing Mirror

**Published:** 1893-12-23

**Authors:** 


"The Hospital" Nursing Mirror.

Being the Extra Nursing Supplement of "The Hospital" Newspaper.

[Contributions for this Supplement should be addressed to the Editor, The Hospital, 428, Strand, London, W.C., and should have the word "Nursing" plainly written in left-hand top corner of the envelope.]

### News from the Nursing World.

### OUR PRINCESS.

All nurses, and especially the members of the Royal National Pension Fund, rejoice to know that H.R.H. the Princess of Wales is recovering from the attack of influenza from which she has been suffering. We wish we could give to "Our Princess" any adequate idea of the volume of sympathetic correspondence which has reached us, and convey to her also a tithe of the kindly loyalty of which she is the recipient. We feel sure that all our readers will unite in hearty good wishes for her Royal Highness's complete recovery to enjoy a Happy and Prosperous New Year with all members of the Royal Family.

### CHRISTMAS GREETINGS.

We receive so many kind wishes for the future prosperity of The Hospital, and so many congratulations on the steady progress made by it since its first public appearance, that we beg to say "Thank you" to our appreciative readers, and also to wish them a Happy New Year. Yes, many, many thanks for all good wishes and friendly Christmas greetings. Each is cordially welcomed and reciprocated. We feel sure that our readers will believe that their favourite journal has every intention "to live up to" the reputation which it has acquired.

### CHRISTMAS FESTIVITIES.

We beg to remind our readers that due notice of festivities and descriptions intended for insertion in The Hospital must reach the office in good time. We are delighted to spare space in our columns for chronicling as many as possible of the entertainments given to patients and nurses, but naturally they are only suitable for the Christmas season, and we cannot permit them to exceed certain definite limits. It will therefore be of mutual advantage if all members of our large circle of correspondents will aid us by sending in their contributions immediately after each event. Any approaching festivities of which preliminary announcements are desired must be officially reported to us ten days at least before they are to take place.

### PRECARIOUS PLEASURES.

We think most people holding authority in institutions willingly help to give the invalids "a good time." Of course, some committees are more liberally disposed than others in taking the initiative in this matter. There are still some places where most of the pleasant adjuncts of Christmas are entirely provided out of the head nurse's limited salary, with such additions as the medical staff or probationers may volunteer. Surely in these progressive times there cannot be many hospitals such as one, with only ten beds, where we hear the committee cannot do anything for the patients, so an appeal for help to give the sick some Christmas gifts is made to The Hospital! There is plenty of kind aid to be secured locally in country districts, if those in authority would take the trouble to bring the patients' requirements to the notice of the inhabitants some weeks in advance of the festival.

### QUEEN'S NURSES IN SCOTLAND.

Rev. Dr. Cameron Lees presided at the fifth annual meeting of the Scotch Branch of the Queen Victoria Jubilee Institute. Ten new local associations have been formed during the year, making in all forty-five, which are now affiliated with the Scotch Branch. The work of the Queen's Nurses appears to be highly valued by the sick poor, to whom their services are freely given.

### A DULL PROSPECT.

This Christmas will be a dull one for the patients in the Edinburgh Royal Infirmary, if the managers keep to their resolution reported in the *British Medical Journal*: "The usual treats or tea parties given in the various wards in celebration of Christmas and the New Year are not to take place this year."

### A NEEDFUL ADDITION.

The Guardians at Bethnal Green have been going into nursing matters, and a proposal for six temporary nurses to be engaged was supplemented by a suggestion to add half-a-dozen permanent workers to the staff. It is to be hoped that the House Committee, to whom the matter is referred, will decide to get fully-trained nurses, and to provide for them suitable accommodation as well as adequate salaries.

### INCURABLES WELL CARED FOR.

In March of this year a little hospital was opened at Bradford under the descriptive title of "St. Catherine's Home for Cancer and Incurables," and already the committee have laid an excellent report before the public. Twenty-seven patients have been nursed, and what "nursing" means for such sorely afflicted invalids most of our readers can guess. The cases are of a kind to demand the most constant and skilled attention, and this has wisely been secured. Great pains were taken in the selection of a Matron, and the committee have every reason to be contented with their choice of Miss Hadley-Scott. At the first annual meeting they "gratefully acknowledged that the devotion and untiring care and patience" of Miss Hadley-Scott and her sister "had smoothed many difficulties, and they had been equal to every demand made upon them." We may well congratulate the Matron on the sympathetic and energetic committee with which she has cast in her lot. The financial report is most satisfactory, and many presents have been received to supplement the diets of patients, whose appetites are probably as capricious as they are limited. Mrs. Rabagliati, the Hon. Secretary, has cause to be proud of the first report issued, and we trust many years of prosperity lie before this excellent little home. After the meeting a large number of visitors inspected the house, and expressed much satisfaction with the arrangements.

### Christmas Books.

"We have passed Christmas without a ghost story. This is not as it should be," observes the learned Miss Gryll in Peacock's novel, and the remark is prelude to a weird chapter on classical and mediæval spooks.

No one is likely to echo Miss Gryll's complaint in the present day. Never were ghosts more tenderly encouraged, and never were they more universally welcomed under any guise they liked to assume. Some few years ago an author in touching on the supernatural felt bound to furnish some loophole of escape which might clear him from any imputation of credulity. Scott ruined "The Monastery" by introducing sceptical passages about the charming apparitions he had created; he could not rest easy under the misgiving that the reader might imagine he believed in them. But the essence of a good ghost story is the air of good faith with which it is told. The narrator may disregard every law of probability, but he will succeed in making your flesh creep if you see he has been really frightened himself. Mrs. Croker has gone on this principle in her clever volume of Anglo-Indian tales, entitled "To Let," and nothing can be suggested more suitable for one of those Christmas evenings which, according to the before-mentioned Miss Gryll, should be devoted to *merveilleuses histoires recontées autour du foyer*. The sketches of Anglo-Indian life are so plainly drawn from life, so full of actuality—to use a cant phrase—that the reader finds himself drawn on without an effort into the region of sinister blue glares, cold icy blasts, dogs bristling like a porcupine, awful, despairing cries, and faint blue torches held by invisible hands, under the impression that it is all going to be cleared up in a minute. But nothing is ever cleared up in this volume, not even the fate of the beautiful "Mrs. Raymond," which, without even a touch of the supernatural, makes as weird an impression as anything in the book. Mrs. Croker, indeed, does not by any means depend upon ghosts for her effects; it might almost be said she interests in spite of them, and her stories may well find acceptance even with that unhappy class of persons who pride themselves on not liking nonsense.

"Our Ghosts," by Edmund Leigh, is a very deceptive title to a book which is neither fish, flesh, fowl, nor good red-herring. It is neither fact nor fiction, poetry nor prose. What it is exactly would be a more difficult matter to pronounce. Perhaps "pretty" is the adjective which best fits in, but Mr. Leigh would do well in future works to supply a more substantial background to his fancies.

We are still not quite out of the region of the supernatural in Mr. Besant's romance of "St. Katherine's by the Tower," which will be welcomed in its new and cheaper edition. The author has taken an old-fashioned case from the end of last century of a girl bewitched into loathing her lover by his hated rival, and relates it from the point of view of a contemporary. It is such a story as may often be encountered in outline in the old witchcraft trials, and Mr. Besant has not spoilt it by any note of superior discernment. Adorned with the topographical details which he has always at his command, and with his intimate knowledge of the period, it makes a very fine story, and the interest, very absorbing in parts, is well sustained to the end.

"Rujub the Juggler" is a dashing story in Mr. Henty's usual spirited style, of travel, adventure, and magic, in connection especially with the outbreak of the Indian Mutiny. Some of the feats performed by Rujub are similar to those repeatedly attested by trustworthy witnesses as performed by Indian jugglers in their presence, and are as uncanny as anything which the most restless imagination could invent. The rope trick, in which a rope is thrown high into the air and stands up perfectly stiff while a boy climbs up it out of sight has never been explained, nor the illusion by which a girl, seated on a block of wood, rises on it as it grows apparently to a tall pole to a distance of 150 feet from the ground before disappearing. The only attempt at a solution of these and similar mysteries of Indian magic, so far as I am aware, is that suggested by Mr. Lang, who divines that the spectators are hypnotised by the juggler into seeing what he pleases. There is some show of probability in this from the fact that occasionally one of the bystanders has been known to protest that he saw nothing whatever.

In "The True Story Book," edited by Andrew Lang, the domain of bogles, witchcraft, and magic is left far behind. The editor, indeed, apologises at the outset for offering a collection of adventures which actually happened to real people. We fancy the number of children who will like it all the better on that account is larger than he supposes. Every narrator knows the sensation of being pulled up short after exhausting his marvel to a juvenile audience by the awkward question, "But is it true?" There is plenty of material in the "True Story Book" to feed the most ardent lover of heroism, and at the same time satisfy the most literal-minded lover of facts. One of the least well known, yet most romantic of the collection, is "The Conquest of Montezuma's Empire," and from this we quote the following spirited passage: "Gunpowder had to be manufactured, and a cavalier, named Francis Montaño, undertook the perilous task of obtaining sulphur for the purpose from the terrible volcano of Popocatepetl. He set out with four comrades, and after some days' journeying, they reached the dense forest which covered the base of the mountain, and forcing their way upward, came by degrees to a more open region. As they neared the top the track ended, and they had to climb as best they could over the black glazed surface of the lava, which having issued from the crater in a boiling flood, had risen into a thousand odd forms wherever it met with any obstacle, and continually impeded their progress. After this they arrived at the region of perpetual snow, which increased their difficulties, the treacherous ice giving way at every step, so that many times they narrowly escaped falling into the frozen chasm that yawned all round them. At last, however, they reached the mouth of the crater, and crawling cautiously to the very edge, peered down into its gloomy depths. At the bottom of the abyss, which seemed to them to go down to the very heart of the earth, a lurid flame burned sullenly, sending up a sulphurous steam which, cooling as it rose, fell again in showers upon the sides of the cavity. Into this one of the brave explorers had to descend, and when they had cast lots the choice fell upon Montaño himself. His preparations were soon made, and his companions lowered him in a basket into the horrible chasm to a depth of four hundred feet, and there as he hung, he scraped the sulphur from the side of the crater, descending again and again until he had procured enough for the wants of the army, with which they returned triumphantly to Hascala." The text is well illustrated by numerous excellent pictures which embellish almost every page.

"Westward with Columbus," by Gordon Stables, R.N., is a stirring record of the trials and triumphs of the Italian hero. Dr. Stables has amplified the narrative to the size of a substantial volume, but he has not abandoned historical accuracy more than could be helped in lending the fresh interest of conversation and detail to the old records. There is a tendency in the admiral and even in the seamen to drop into polysyllables when anything out of common is afoot, but on the whole the story goes ahead with ease and vigour, and will no doubt be very popular both as gift book and reward. A serious defect is the want of any map or plan to assist in making clear the numerous land wanderings of Columbus, or the points on the westward coast at which he touched. In subsequent editions this want should certainly be supplied.

### Help the Nurses to Help the Sick.

"A LITTLE HELP IS WORTH A DEAL OF PITY."

YES, yes, it's very true, and very clear,
By way of compliment and common chat,
It's very well to wish me a New Year,
But wish me a new hat!

Although not spent in luxury and ease,
In course a longer life I won't refuse;
But while you're wishing, wish me, if you please,
A newer pair of shoes!

Nay, while new things and wishes are afloat,
I own to one that I should not rebut—
Instead of this old rent, to have a coat,
With more of the New Cut!

Oh yes, 'tis very pleasant, though I'm poor,
To hear the steeple make that merry din;
Except I wish one bell was at the door,
To ring new trousers in!

To be alive is very nice indeed,
Although another year at last departs;
Only with twelve new months, I rather need
A dozen of new shirts.

Yes, yes, it's very true, and very clear,
By way of compliment and common chat,
It's very well to wish me a New Year,
But wish me a new hat! —*Tom Hood.*

There are many ways in which this "helping" can be done. By donations to useful charities, or by annual subscriptions, and, again, by giving to individual sick persons the things most needed, such as skilled nursing, good nourishment, clothing, &c. We must all do something, especially at Christmas, for the sick and suffering—not grudgingly nor of necessity, but willingly and gladly, feeling the blessing and privilege of giving, and not looking for present thanks or rewards.

We see gratitude very often so disproportionate to small offerings that the donors must feel humiliated as well as encouraged to further efforts.

It is rather the fashion to say that workers in hospitals have more opportunities of doing good than those living in the outside world. But the latter can help to increase the usefulness of the former to an unlimited extent. Convalescent homes for discharged patients have to be provided; railway fares must be defrayed; warm clothes of all sorts have to be found for men, women, and children who have recently recovered from dangerous or painful sickness. Sound boots and warm stockings are sorely needed at all times for the hospital patients who quit comfortable wards for wet pavements, and who miss the food and fires which they have enjoyed most keenly when they have to relinquish them.

Those whom home duties or other reasons keep away from sick wards can still help immensely to brighten these. They can send flowers, not merely big bunches of summer blossoms when everybody has plenty, but winter posies to rejoice the eyes of the sick. Palms, indiarubber, and many such plants live, yea, they even grow and thrive in institution life. They get such loving care, and each new leaf receives such a joyful welcome, that every sick person's eyes should be refreshed by the sight of some green-growing plant within view. A plant, a bundle of socks, shoes, shabby if sound garments, all are welcome by busy head nurses, who know well the needs of the patients whom they serve so well and cheerfully.

We give the names of a few of the numerous good works which appeal for help and encouragement to all who are interested in their poorer brethren.

**Queen Victoria Jubilee Institute.**—The headquarters of the Institute are at St. Katherine's Hospital, Regent's Park, and information can there be obtained from Miss Peter, the inspector. Only fully-trained women are enrolled as Queen's nurses, and the demand for them in country as well as metropolitan districts increases annually. There are Scotch, Irish, and Welsh Branches of the Queen Victoria Jubilee Institute.

**Workhouse Infirmary Nursing Association.**—Much progress is being made in introducing an improved system of nursing into workhouse infirmaries, but much yet remains to be done. Trained nursing is making its way surely, if slowly, and the recommendation of Dr. Downes, the General Medical Inspector of the Local Government Board, that pauper nursing be entirely abolished, should have good results in the near future. Increased financial support is urgently pleaded for to extend the work of training probationers, which is the object to which the greater part of the funds are at present applied. The objects of the Association are: To raise the standard of public opinion on the whole question of workhouse nursing; to secure the appointment of trained ladies as matrons in all separate infirmaries; and to train and supply nurses to workhouse infirmaries in London and the provinces. H.R.H. Princess Mary Adelaide of Teck is President of the Association, and subscriptions will be gladly received by the Hon. Secretary, Miss Wilson, 13, Barkston Mansions, S.W.; or they can be paid in to the account of the W.I.N.A., at Lloyd's Bank, 215, Strand, London.

**Samaritan Societies.**—The work done by the Samaritan societies connected with our hospitals can hardly be overrated. The help afforded by them to poor and destitute patients is of the utmost value. When, owing to the demand upon hospital accommodation, cases are reluctantly dismissed before normal health is recovered, then the succour provided in the way of clothing, admission to convalescent homes, surgical appliances, or whatever may be needed to carry on the treatment already received in the hospital ward, is of untold value. Funds are always much needed for this most useful work, which is, to a certain extent, perhaps, somewhat overlooked amongst the ordinary charitable appeals. We would impress upon our readers that in contributing to these societies the work of the hospitals is very practically assisted.

**The Royal National Pension Fund for Nurses, 8, King Street, E C.**—This society is prospering exceedingly, and its valuable work is becoming more widely known and appreciated every day. Those who failed at first to fully comprehend its advantages, have found the objections ignorantly advanced have entirely disappeared in the light of wider knowledge. A new scheme is in course of completion, by which a nurse, for the payment of the small monthly sum of 3d. or 6d., is assured of £5 to £10 at her death, if it occurs after she has entered upon her pension. This is to meet an often-raised argument against entering the Fund, namely, that should a nurse die a few years after she has begun to receive her pension, her friends cannot get back any part of the money paid in. The Benevolent Fund Branch of the Royal National Pension Fund continues to do good work, affording substantial help in case of long or short illnesses, and consequent incapacity for work.

**The East London Nursing Society.**—Since the establishment of the East London Nursing Society, some four-and-twenty years ago, the sphere of its labours has largely increased. Its nurses work in twenty-nine East-end parishes, and their helpful ministrations are sadly needed in these overcrowded districts, as may be gathered from the figures in the annual report showing the number of cases visited during the year. From January to October, 1893, 3,728 persons were nursed and helped. A larger permanent income is much needed for a further increase of staff, and we sincerely hope the necessary funds may be promptly forthcoming. Those wishing to help should communicate with the Secretary, Mr. Arthur W. Lacey, 29, Philpot Street, Commercial Road, E.C.

**East End Mothers' Home.**—As the only Maternity Hospital in the East-end district of London, this Home should appeal successfully for increased help to all charitable people. It has only twelve beds, and the demand on these is great and ever-increasing. The out-patient department supplies skilled attendance to mothers in their own homes, and greatly needs extension to meet the needs of the growing population. Help will be gratefully received by the Secretary, Mr. A. W. Lacey, Swiss Cottage, Dacre Park, Lee, S.E.

**Royal Sea Bathing Infirmary at Margate.**—This unique and valuable institution is so badly supported that many of the fine wards have been reluctantly closed. It is grievous to think of the empty beds in those noble rooms, when so many patients might there be restored to health and usefulness. It is the only hospital in England set apart exclusively for scrofula, and the cures which result from a lengthened stay are indeed remarkable. Of all the institutions which call for liberal support from English men and women this fine hospital is one of the most worthy. All information will be gladly given by the Secretary, Mr. Arthur Peirce, 30, Charing Cross.

**The Invalid Children's Aid Association.**—This ideal charity has its offices at 18, Buckingham Street, Strand. The object of the Association is to help invalid and crippled children in their own homes. A system of regular visiting is organised, and the ladies who volunteer for this work find out what is most needed by the little people, and endeavour to supply it. Spinal carriages, perambulators, surgical instruments, and bed linen are lent, and clothes are often given; also toys, picture books, &c. Tickets for seaside and country convalescent homes are distributed—in fact, much useful work is unobtrusively done by this Association. Subscriptions are gladly received by the Hon. Secretary, Mr. Allen Graham.

**Up-Country Association for Europeans in India.**—This Association is designed to meet a much-felt want, and to supply trained nurses to up-country Indian stations, where their services are too often in urgent request. Many Europeans in these out-of-the-way stations are quite unable to afford the heavy expenses of having a nurse sent up-country, even should one be obtainable, which is not always the case, the supply of trained English nurses in India being quite unequal to meet the demand for them. No new organisation is contemplated by this Association, its purpose being to enable existing institutions to add to the number of their workers. The committee estimate that £50 will suffice to export and establish one nurse in India, to be placed "within a reasonable distance, at a reasonable cost," for future patients. The Queen-Empress and also the Duchess of Connaught are particularly interested in the scheme, of which further particulars can be obtained from the Hon. Treasurer, the Hon. Mrs. Neville Lyttelton, 21, Carlton House Terrace.

### Our Needlework Competition.

Once more the pleasant task of unpacking Christmas parcels has fallen to our lot. Packages of various shapes and of all sizes have arrived, and met with a hearty welcome.

If they were a hundred times as numerous they would be gladly received, for so many appeals come to us in the winter for socks, flannels, and garments of all sorts. Sometimes we find it very hard to make reply, "We've really nothing for you."

To those amongst whom our stores can be divided it is easy enough to write and say a parcel is on its way. Those friends whom the supply this year is inadequate to reach must take heart and believe that their turn will come next Christmas.

The prizes have been adjudged as follows: (1) The best pair of socks knitted by a nurse, 5s., to Miss Elphick; (2) for the best pair of socks knitted by any Hospital reader, 5s., to Miss Cotterill; (3) for the best made flannel shirt, 10s., to Nurse Clark; (4) for the best flannel or flannelette bed-jacket, 10s., to Miss Constance Delap; (5) for the best flannel petticoat, 10s., to Miss Mary Delap; and (6) for the best made and simplest-shaped dressing gown made and cut out by a nurse, 20s., to Sister Lucie.

A most beautiful nightdress, with stitches in the most perfect order possible, has received great admiration. It was made by Miss Weston. The dressing gowns were also particularly good this year, and the prize flannel petticoat is a model of excellent work. There is another flannel petticoat which is highly commended. Nurse Cooper must have taken great pains with it.

We have received flannel shirts from Nurses Clark and Elizabeth Bishop, and Miss Constance Delap; bed-jackets from Miss Delap and Nurse Smith; flannel petticoats from Miss Mary Delap, Nurse Cooper, Miss Janet Mundy, Miss Blann, and Miss Lockyer; dressing gowns from Sister Lucie, Nurse Sherring, and Nurse Francis; and without any address, knitted vests, children's stockings, grey petticoats, comforters, bed socks, man's jersey, all very nice; knitted socks from Miss Janet Mundy, Miss White, Nurse Annie Cole, Miss Elphick, Nurse Esmee, Nurse M. Griffiths, Miss Alice Cotterell, Miss Hunt, Miss E. Fletcher, Nurse Anderson, Miss Lomax, Miss Child, Miss M. Simpson, Miss Mabel Allen, Miss C. Delap, Miss Chisnall, Miss Disney, and Miss R. H. M. Smith.

The socks and stockings are many of them excellently knitted, and they are most acceptable and valued gifts.

We have just sent off parcels to King's College Hospital, the Middlesex, Charing Cross, the London, the Royal Free, and the Westminster and St. Thomas's Hospitals.

### Christmas Day.

### THE DISTRICT NURSE.

A district nurse's Christmas Day, especially in London, is rather wanting in the effect of brightness and cheerfulness which characterises that of her sister workers in the hospitals. So many of the homes she visits on that morning are clouded by the hopeless out-of-work poverty which is apt to deaden all feeling beyond the struggle to feed the children and keep the home together. When the breadwinner or the mother is on a bed of sickness, which means less coming in and more being spent, it is difficult to rouse feelings for a "Happy Christmas." Still, the majority of the people do make an effort to brighten their dingy rooms a little. Evergreens and holly are arranged in various ways, and long festoons of links of coloured paper are suspended from the middle of the ceiling, radiating like spokes of a wheel to the sides of the room. The effect is bright and pretty, though trying to tall visitors. This kind of decoration is most popular among the coster class.

Thanks to the numerous charities of the season, many a household has a good fire and a Christmas dinner for once, and generally some kind of a pudding is made "for the children." A nurse can always reach the children, at any rate, and give them a share in what is so specially the children's festival. A suggestion for stockings to be put out for Santa Claus to fill will keep little minds in happy excitement for days beforehand. Friends can always be found who will contribute small and suitable gifts for the patients at this time, and it is pleasant work arranging them beforehand. Each nurse knows what is wanted by those under her care. The warm shawl for the old lady just recovering from bronchitis; the worn and mended, though still warm, flannel shirts for the man just convalescent from rheumatic fever; the comfortable knitted vests for a child lately at death's door with inflammation of the lungs—all these are tied up and labelled beforehand. Of necessity, many are distributed the day before, with strict injunctions not to open the parcels till the next morning.

The nurse's work begins an hour later than usual, like Sunday, but by half-past nine she has started on her round, carrying some of the parcels with her. As far as possible only the severe cases are seen on Christmas Day, for many of the chronic ones have friends with them only too pleased to render what help they may need. Still even this means many visits for the nurse to pay, as there is always much sickness amongst the poor in winter. The faces brighten as she enters with the familiar greeting, and the Christmas wishes are heartily exchanged.

It is generally close on dinner-time before all are visited, though at the end of her round the nurse's burdens have visibly decreased, and each home is a little brighter for her presence in it. After a quiet afternoon the evening work begins at five p.m., for temperatures must be recorded and poultices renewed, though in every case patients' friends do all they can "to save the nurse" on Christmas Day. Generally she can spend the remaining hours with friends as her share of the day's pleasure, but in district work it is always uncertain when new cases will be sent, and many a pleasant plan has to be laid aside at the last moment.

### Christmas Day With a Private Case.

By a Nurse.

Looking back to past Christmasses, one particular anniversary stands out in my mind clear and distinct from all others. Why it should be so I hardly know, for December has been an eventful month in my life, bringing sometimes keen sorrows, and, oftener still, blessings and peace.

However, the special Christmas Day I am thinking of now had certainly no great sadness, and a vast deal of variety about it! I had returned from my last case two days before, and Matron said to me when I arrived at the Home, "Nurse, I think you had better take a day or two's rest, I won't send you out again until after Christmas if I can help it. But there's so much sickness that I dare not promise positively," and she sighed, I remember, as she poured me out a hot cup of tea.

I had two delightful quiet, restful days and was fain then to announce myself perfectly fit for work. This was lucky, for an imperative summons arrived on Christmas Eve. It was a doctor who brought it, and he hadn't a moment to spare, but just gave me rapid orders and explained, "It's a young lady, nurse. Sharp attack of bronchitis, only just moved into a new house; very uncomfortable for you, I expect, but they seem nice people. Can you go at once?"

The house was a fairly large one, standing at a corner where three roads met. I opened the gate, and followed the footpath which led to a flight of wide steps, and on these I encountered a tall young man. He looked at me in the moonlight and exclaimed, "I'm thankful to see you nurse. How quick you've been. I'm afraid you will be disgusted with our establishment, but I'll have things more comfortable presently." Then he opened the door with a latch-key, and by the light of the flaring hall lamp I saw he had an empty coal box in his hand. Noticing my look of surprise, he explained, "We ordered coal to come in a week back, but the state of the roads is made the excuse for the delay. However, a man's bringing in a couple of sacks for us presently, and I'm going to borrow a boxful to go on with. They lent us some next door this morning."

It was a comical scene on which I had entered, and I began thoroughly to enjoy it; I liked the unaffected young man who had taken the burden of discomfort so cheerfully on to himself. In a large bare room lay my patient, a small iron bedstead was placed in front of the fire, and a tall clothes horse had been improvised into a screen by the addition of two blankets. There was a grand fire but no fender, and a piece of a walking-stick had done duty for a poker. A boy about fourteen was stirring something in a saucepan, and looking rather flushed as I came quietly round the screen.

"Oh, I say," he exclaimed in a tone of relief, "perhaps you can tell me if this is nearly done? It makes Ailsie cough to talk, and Jack said I was not to speak to her, but to mind and keep this from boiling over, but it seems getting awfully lumpy!"

I lifted the saucepan and found some very satisfactory arrowroot in course of thickening. "Yes, that's all right, but did the doctor order it?" I inquired.

"He said she could have any milk stuff she fancied, and Jack said she used to make some of this when he was ill once, so he started cooking it."

I moved the light, which was flaring in Ailsie's eyes, and put it a little behind her, and she thanked me with a grateful look, for a fit of coughing prevented her speaking. Then I found a cupboard on the landing, into which I put bonnet and cloak, and returning speedily in cap and apron, set myself to work. The patient certainly had a sharp attack of bronchitis, and she must have kept about in the cold, bare house when she ought to have been in bed.

But when I had carried out the doctor's orders as to outward applications and inward remedies she grew easier, and I left her in young Donald's charge whilst I went downstairs to collect such things as might be needed during the night. Most of the rooms had the furniture that belonged to them deposited in the middle of the floor, evidently just as the men had placed it. A pleasant breakfast-room next to the kitchen was in fair order, and the boys' bed-rooms also.

The tall young man, who proved to be a mere lad of seventeen or eighteen, was very busy in the kitchen. He had taken off his coat and pinned a large piece of wrapper round him apronwise, and looked full of importance.

"How do you think Ailsie is?" he asked, immediately he heard my footstep.

"I think she will get on all right if she is well looked after for a few days. You and your brother are good nurses."

He gave a pleased smile, and then his face grew sad as he answered, "Unluckily we have had a good bit of experience, nurse. As for Ailsie, she's just knocked herself up. We had to move rather suddenly because the drains were all wrong, and our housemaid was ill with a bad throat. So Ailsie nursed her up and then took her over to an aunt at Balham. We thought she might be all right in another week, and we could manage with the cook. She's a real good sort, I can tell you, but she slipped on a slide yesterday and twisted her ankle, and she's keeping her foot up in the breakfast-room. I'm afraid you'll want to run away from us. We're in such a muddle!"

But I didn't run away. The cook was a dear old soul, shedding tears over her helplessness. When the doctor came in last thing at night he helped us to move the poor woman upstairs, and we made up a couch in Miss Ailsie's room, so that I could look after her at night, and she acted as watcher by day, and rang when the patient wanted my help. What fun we had; and, oh! how I worked helping them to get straight. On Christmas morning Jack made an omelet for breakfast. It was really delicious; he cooked it in a jam-tart tin, and he brought it to the table in triumph. He had made himself a white paper cap like a *chef*, and with his good-tempered jests helped to keep up all our spirits.

The water pipes burst during the day in three different places, and we had some trouble in stopping the leaks. Of course all the plumbers were "keeping Christmas," so we did not waste time in hunting for them. Then the kitchen chimney smoked, and the oven would not heat, so we decided perforce to dine late. And we filled up the interval with a little unpacking and arranging the patient's room.

"I am so much easier," said Ailsie to me that night. "I felt very bad before you came; in fact, I was beginning to wonder who would get things straight for the boys if I died. Aren't they handy boys?" she asked, with a proud affection, and I gave a willing affirmative as they entered her room.

"Now, Ailsie, we've had a proper Christmas dinner, thanks to nurse, and we want to sit round your fire and feel Christmassy." Then, added Jack, turning to me—

"Are you really a trained nurse?" and I responded.

"Yes; don't you think I'm a good one?"

The boys both blushed a little, and Donald blurted out—

"To tell you the truth, we begged the doctor not to get Ailsie a regular nurse, because we thought she'd be a nuisance, but, thank goodness, you are quite the reverse. In fact, old cookie calls you, 'The real good sort,' behind your back!"

### Appointments.

Wallsend, Wellington Quay, and Howdon Joint Hospital.—Miss Mary Crathorne has been appointed Matron of this hospital. She was trained at the Infirmary and Dispensary at Bolton, and was for two years Nurse at the Hull Nurses' Home, for a short period Sister at the Hull Sanatorium, and Head Nurse at the Crosland Moor Union Infirmary, Huddersfield. Many good wishes accompany Miss Crawthorne in her new work.

City Hospital, Parkhill, Liverpool.—Mrs. Kate Daly has been appointed Matron of this hospital. She was trained at Brownlow Hill Parish Infirmary, and was afterwards Superintendent of Nurses at Bradford Fever Hospital, and Matron of Bootle Fever Hospital. We wish Mrs. Daly all success in her new work.

### Christmas at Biarritz.

"I have to go to Bayonne this evening; will you come with me?" asked a pleasant voice with a slight French accent.

It was Christmas Eve, and I had been spending the whole day in a sick room, with a rather nervous invalid, so the invitation sounded particularly tempting. I was "off duty," too, and my patient was in competent hands, so I did not hesitate for a moment.

Things may be changed now, for it is some years since that pleasant evening when we stepped into the little "omnibus train" which conveyed us from beautiful Biarritz to picturesque Bayonne. A quiet, steady-going affair was that small train, and we had plenty of time to enjoy the clear sky and soft air as we passed along. There was something exhilarating about this "evening out," for I had not been long enough in the neighbourhood for the place to have lost any of the charm of novelty. The shops and booths and stalls were never-ending amusements, and the bright lights and colours everywhere formed a wondrous contrast to December hues in London.

We did a little shopping and a great deal of looking about, my French companion pointing out different objects, and sketching off the histories of some of the citizens whom we encountered, with graphic words and gestures.

We went into a confectioner's and had some fascinating cakes, which left us still hungry, though too thirsty to go on eating.

We returned on the roof of the railway carriage, enjoying the light wind which swept across from the mysterious looking sea. How quiet it seemed, almost motionless, save for the dainty "white horses" which danced on the "bar." Treacherous waves for all their fairness, and many a good boat has come to grief at that notorious spot.

It was a never-ending interest at Biarritz to watch for the changes in the sea. Walking quietly on a fine day we would suddenly notice the waves rising, and in an hour they would be dashing high in the air, thundering over the rocks and sending spray far over the land. Yet the bright sunshine and cloudless sky seemed to mock at the wind storms which troubled the ocean.

But such storms appeared impossible the night we travelled slowly back from Bayonne on the omnibus train. On arrival the inevitable search was made at the station barrier, and smiling housewives shrugged their comely shoulders whilst demanding how monsieur could suspect them of evading the tax? Poor souls, it seemed hard that the chicken or quart of milk they had purchased must if "declared" or discovered pay duty so strictly.

"The night nurse says, will you please relieve her a little earlier to-morrow, as she is not very well," was the greeting I received on re-entering the villa. So I changed my light cloak for an apron and went to see what was the matter; and the end of it was that, after a hasty cup of tea and a piece of white bread, I just went on duty again and sent my co-worker to bed, trusting sincerely that a night's rest might set her to rights and ward off the sick headache to which she had succumbed.

Christmas Eve! I remember now how I sat and nursed up that wood fire, striving to keep it in without disturbing the light sleeper I was watching. I thought of many past "Eves," some happy, some sad, all busy ones. This winter I had been persuaded to go abroad with a patient, and it sounded attractive and restful, so I went. It speaks well for the air of Biarritz that I really enjoyed the place, although the people were rather depressing. But the patient improved, and I sometimes think she took a turn for the better during that lovely, unfamiliar "Eve." Sunshine of the most brilliant description heralded in the great festival, and early in the morning, "A Happy Christmas," said a quiet voice, and "I have had a good night's sleep at last, dear nurse; give me my chocolate, and tell me I'll get better and try your loving patience less."

H. F. G.

### Christmas in a Workhouse Infirmary.

By an Infirmary Nurse.

What a busy day it is, to be sure, for the nurses, but how cheerful everything looks, and how bright and gay are our patients! Perhaps nurses in a general hospital say, "Can any good thing be in a workhouse infirmary?" But, at least, let me tell about the way we spend Christmas in our own special Infirmary. Matron, charge nurses, and probationers rise at half-past five, and we are nearly one hundred altogether.

We begin by partaking of the Holy Communion, this is not, of course, compulsory; breakfast at half-past six, and each nurse finds on her plate a Christmas greeting from the matron. We go into our wards at seven punctually, relieving the night nurses, and are greeted with shouts of "A merry Christmas, nurse!" from numerous patients. No acute cases? Oh, yes, there are plenty. It is very hard work to get our patients and wards ready for the doctor's visit at ten a.m., but it is done.

After his visit the preparation for dinner begins, almost all may have roast beef and plum pudding, and the table for the convalescents is beautifully decorated with flowers and fruit, and the trays for those unable to rise are most extraordinarily highly polished, each charge nurse trying to excel her neighbour. Dinner is carved and served out, and, when all is cleared away, amusement begins. Patients who are well enough are allowed to visit other wards. So are the nurses, one being left on duty. Many visits are paid, and the decorations are freely criticised.

Some wards are much more tastefully decorated than others, and on the whole, the male are best. On the other hand, the women patients are more picturesque than the men—dressed in blue gowns with scarlet cloaks and white caps they are quite attractive. The men come over to the female side, and the floor is certain to be the first object remarked upon. Our ward floor is really beautifully polished, and the men exclaim "Why, nurse, who polishes for you? This is almost as good as ours!" The children's ward is gay with a hugh Christmas tree, of course, and by the time we all return to our wards it is tea time.

After tea the fun begins, for we have then a piano. The doctors come up, and one accompanies a nurse's song while another plays the violin. "Fiddle and I" is performed, &c. Then another doctor, who thinks he can sing, gives a ditty which takes remarkably well, all the patients joining in the refrain. This is encored by all, and we have next a pathetic song, "The Miner's Dream." Then the musicians go on to another ward and repeat their entertainment. But this is not the only concert, for half a dozen are going on in the different wards.

At seven p.m. there is a grand concert and tableaux vivants given by the nurses, to which all who are able to attend may go. Great excitement prevails when a patient discovers his or her own nurse in disguise. Also "our" nurse is always considered to sing better than anybody else! Concert and tableaux are at length over and the patients wend their way back to their several wards very tired, happy, and sleepy. They are all tucked in and left in possession of the night nurses.

Peace and goodwill reign around, and the nurses, weary indeed, are yet happy in the knowledge that they have given to 1,400 patients a merry Christmas Day in the workhouse infirmary wards.

### Nellie's Christmas.

Christmas carols, ringing sweetly,
From the beds and round the tree;
Carols sung by children's voices,
Some but weak, yet full of glee!

Tiny little red-clad figures,
Stretching out their wasted hands,
While the Christmas tree, all lighted,
Crowned with gifts in glory stands.

Only one small voice was silent,
One small face was full of care;
Such a weary little maiden,
Paler than the palest there.

All the nurses loved to please her,
Tried to cheer her as they passed;
For they knew (altho' *she* did not)
That this Christmas was her last.

One, at length, beside her pausing,
Asked her, "Nellie, why so sad?
Don't you like your Christmas presents?
See the others all are glad."

Nellie answered, soft and slowly,
"Yes, I'm glad and warm to-day;
But I can't but think this Christmas
Will have soon to pass away.

"And next Christmas I'll be hungry,
Cold and weary, aching too;
And have no one more to help me
And to love me as you do.

"Last year, in the snow at Christmas,
I was cold and full of pain;
And 'tis that I can't help thinking—
Will it be like that again?"

And the sister, o'er her leaning,
Bent to soothe her with a kiss,
Whispering, "Childie, your next Christmas
Will be better still than this."

Nellie smiled, with face uplifted,
Trusting, asked not why or how;
Drew her gifts with joy toward her,
Blessed in her Christmas now!

C. H. Hammill.

### Christmas Day in Japan.

By a Traveller.

I once spent a little time with some hospital nurses in the million-peopled capital of Japan. At no season in the year does a traveller feel so entirely a foreigner in a strange land as on Christmas Day in Japan. Although missionaries are countenanced and seldom interfered with, yet Japan remains to-day a Pagan country. Christmas is, therefore, the festival which strangers living in treaty ports and a few converts have all to themselves.

English visitors in Japan try to keep Christmas in the old-fashioned way, and the whole of the foreign settlement looks *en fête*. The Japanese servants do all the decorating, making over each doorway a stiff arch of evergreens; oranges on pointed pieces of stick are thrust into the wreath at regular intervals, and the English and Japanese flags are crossed over the door.

We only allowed the smiling Taki (our porter) to ornament the exterior of the hospital. This formal style of decoration he must have learned along with other mistaken ideas with the motive of pleasing the English. The walls were adorned with plum blossoms, white and pink, which were beginning at that season to colour the land, and also with crazy little fir trees, so dear to the Japanese taste, and great branches of orange trees heavy with fruit. No doubt for all practical purposes these would have served as well as the time-honoured mistletoe. The plum blossom is really at its best by the end of January, but at Christmas you can buy in pots the little old plum trees, a few inches high, knotted and twisted by years of careful tendance into a hundred fantastic angles. We had bright Japanese lanterns suspended from the branches of these dwarfed shrubs, and in the dim light the long lanterns and distorted branches threw delightful shadows on the white ward walls. Some out-patients for whom we had done trifling kindnesses came in to help us, and when our native friend Mayeda San arrived in the evening he expressed his entire approval by the more than usual drawing-in of his breath with an inward "hiss," and by countless rubbings of his knees. He brought us each a present tied up in the proper shape with red and white paper strings, and a bit of dried fish done up in bright paper, the latter always accompanying a present in Japan. My gift proved to be a little velvet musk-band, with a quaint silver clasp, which Japanese ladies wear on their arms in a crowded room in lieu of a scent bottle. To another he gave a small wicker-work case, like a pocket-book; the Japanese ladies carry their paper pocket handkerchiefs in one of these in the long sleeve of their "Kimono."

To Sister L., for whom Mayeda San entertained a more sentimental feeling, he gave a poem, exquisitely written on dainty rice paper, with the design of a shadowy plum tree spreading over it. Poor Mayeda San, I'm afraid the point of the sentiment did not gain full appreciation, as the characters were Chinese. At the English Church we were really reminded of "Christmas Day in England," the sacred edifice was beautifully decorated after the true British fashion, abundance of cotton wool for snow and plenty of sealing-wax for holly. Japan was almost forgotten, and the congregation was enjoying "Hark, the herald angels sing," when the side door was modestly opened and discovered a crowd of smiling Japanese. The ever-important verger at once went forward greatly to their confusion. They had all slipped off their sandals, according to the custom on entering any building, with the intention of getting in unobserved. They had displayed their usual good taste by coming as quietly as possible and if their exit was less graceful and more noisy it was the fault of the verger, for covered with confusion they somehow got mixed in the matter of sandals. After our turkey stuffed with oysters, for it was an American hospital, we went out for a Rikisha drive along the tiny foot tracks which separate the paddy (rice) fields, and past a little brown temple. So poor and bare, a sanctuary where beauty and fashion never entered, but yet some poor Coolie, pausing on his homeward way, might slip his bamboo pole and basket off his shoulder, clap his hands to call the attention of the God in question, and then clasp them for a moment's silent prayer. Clapping again on finishing, he will once more take up his burden and become the typical figure in the fantastic landscape.

The hospital Rikisha-boy asked me to wait (mati) while he offered a prayer at the fox-guarded shrine which we passed in a dark camellia grove. He was a Christian by profession, a regular attendant at the mission, but there was a rice famine in the land and, at the sight of the imaged God of Rice the old belief sprung up, and the expression of his uplifted face showed the prayer was earnest. Somehow Christmas Day had seemed lost in the watery waste of the paddy fields, but as we neared home we passed the native mission church. It was prettily decorated with three Christmas trees on the platform, where the pulpit stood, and the building was full of smiling Sunday-school children and grown folks. They were singing carols and there was a dialogue going on between Santa Claus and some Jap children; though it was all said in Japanese yet I felt the Christian's Christmas Day in that Asiatic congregation.

Earnest and devout as the assembled seemed, I could not help questioning whether, if on their homeward way, some of them had to pass through the long, dark camellia grove, carpeted with fallen crimson blossoms, they might not pause awhile and hold a little monologue before the white stucco foxes of the "God of Rice."

The New Year is the chief annual festival in Japan, and during the week the whole land is full of strange sounds and stranger sights. The first of January is the day of reckoning with the Japanese; all standing debts must be paid on that day, or the debtor is dishonoured.

On New Year's Eve the streets are converted into a "Cheap Jack" bazaar of curios and second-hand articles of astonishing variety, and the scene is illuminated with flaring torches. The sales begin at nine o'clock in the evening, and for the curio hunter it is a night of heavenly bargains. At the same time, it is intensely pathetic. Some poor woman, a mere living skeleton, shivering and half-clad, is offering to the blasé foreigner her last humble household gods. It may be one of Lord Bass's empty bottles, or one of Pear's empty boxes, which have no meaning to her, and for which she asks twice their value when full. Perchance an old blue china flower-pot, with a carefully-dwarfed plum tree, just bursting into pink bloom, may be the bit of real Japan she wants to sell. As the old year dies the lighted torches grow fewer and fewer. In the wind-blown light the street becomes a dark mass of stooping figures, packing up their neglected wares. The clock in the Ginza records the midnight hour (foreign time), and the hungry old woman thinks of the morrow and implores you to take her all for 15 cents.

Then the day breaks and the shadows flee away and all the land is smiling, nothing but pleasure receives any attention for a whole week.

### Christmas Carol.

God rest you, merry gentlemen,
Let nothing you dismay,
Remember Christ, our Saviour,
Was born on Christmas Day.

So runs the old carol, and with thankful hearts we will join in the burst of song which nearly 1,900 years ago first came through the silent night, from angels in heaven and shepherds on earth. However gloomy the end of the year may seem, we know that "at eventide there shall be light." As the twelvemonth dies slowly out the day-spring of our salvation draws nearer and nearer, and we now rejoice in the birth of the Lord of Glory, who comes to us meek and lowly, as the Babe of Bethlehem, lying in the stable manger or pillowed on His mother's breast.

What a wondrously beautiful thing it is to consider the immense glory which is thus reflected on childhood by God's Incarnation! How tenderly should we look on our little ones, who are the very patterns of what our dear Lord once was, types of the Christ who suffered them to be brought to Him in later years, because of such as they is the kingdom of heaven. Happily, this glory is reflected on manhood also. How fully may our hearts ponder in peaceful contentment as we feel that the promised Saviour has come at last in the fulness of the time, that

He has come the broken hearts to bind,
The bleeding souls to cure,
And with the treasures of His grace
To bless the humble poor.

Even our pains and bodily sufferings may for once be swallowed up in gratitude for the birth of our Saviour and our King; they are lost for the time in thinking of God's inexhaustible love which saved mankind. In breathless wonder we follow His footsteps "from the poor manger to the bitter cross," and see how He has consecrated our trials, our pangs of mind and body by bearing the like Himself. Christ made childhood glorious by taking our flesh and dwelling in a humble cot at Nazareth in subjection to His parents. Womanhood gained higher dignity from the Virgin Mary tending her infant Son, just as other loving mothers do to this hour; watching the loved face broaden, and lengthen, putting off and taking on the expressions of the successive ages of human life. She doubtless pressed His sinless face to her own in the liberties of maternal love, so we too may solace His childish tears and feebleness by nursing and tending all infant sufferers. "Insomuch as ye have done it unto one of these little ones, ye have done it unto Me," will be our reward.

Labour has been ennobled since the Son of Man worked in the carpenter's shop, earning the bread that perisheth by the sweat of His brow, and earthly friendship is consecrated by the close affection which subsisted between our Lord and the beloved Apostle.

With our Saviour then we will bear our sufferings in the spirit of His love. We will go on "toiling, rejoicing, sorrowing," until, this life ended, we shall share in the endless Alleluiahs with the redeemed around the throne.

Meanwhile, till we have gained this consummation of bliss we will join the whole creation in praising the Lord with glad hosannas to the Prince of Peace, evermore and evermore.

### How Bobbie Spent Christmas.

Well, 'ere I'm back in London
An' with you all once more,
An' my leg, it's gettin' better
Nor it ever was before.

I've sech a lot to tell yer
Of all the things I've done;
An' best of all at Christmas
My! we did 'ave some fun.

(I was near well at Christmas;
I didn't 'ave no pain.
They said the change 'ad done me good,
An' I could walk again.)

So some of us on crutches
An' some of us in bed,
We sang the Christmas carols,
The nurses stood an' led.

An' after that (you young 'uns
What ain't been there to see,
You just can't think what it was like!)
A monstrous Christmas tree!

There was candles on its branches
An' sweets an' toys an' things;
An', at the top, a fairy,
With lovely golden wings.

An' the candles all was lighted,
A hundred I should say—
An' they kept 'em all a burnin'
Till they burned theirselves away.

An' they gave us all a ticket
An' then we 'ad to wait
To see what we'd get off the tree;
An' mine was number eight.

Then someone took an' brought me
A ball from off the tree,
'Twas such a beauty, that I thought
It couldn't be for me!

An' sweets—ah, they was goodies!
Chocolates—such a size!
Not like the 'a'penny mixture
Wot gen'rally we buys.

My! that was like a Christmas!
An' when I went to bed
I 'ad my ball to sleep by me
On the chair beside my head.

You've not had sech a Christmas!
But then, you kids, you see,
You've never bin to 'orspital
With a bad leg, like me!

I shouldn't mind next winter
If my leg was bad again;
The 'orspital's so comfy that
I don't much mind the pain.

I never had so nice a time
As on that day—not I!
An' I specks that I'll remember it
Till I grow old an' die."

C. M. Hammill.

### The Book World for Women and Nurses.

[We invite Correspondence, Criticism, Enquiries, and Notes on Books likely to interest Women and Nurses. Address, Editor, The Hospital (Nurses' Book World), 428, Strand, W.C.]

### CHRISTMAS BOOKS.

Tales of the Children's Ward. By Honnor Morten and H. F. Gethen. (Sampson Low, Marston, & Co.) 3/6.

This is a delightful volume of stories. Hospital and nursing matters are frequently brought into modern literature, not always perhaps with the best results, and fiction founded on such subjects has often little to recommend it, but these bright and natural sketches of life in the wards of the "East End Hospital" can be productive of nothing but good, by helping to bring into prominence the crying needs of the little ones in our midst. Miss Morten and Miss Gethen write with the heartfelt sympathy born of an intimate knowledge of the wants and woes of sick and suffering children—what terribly sad visions the words call up!—and pathetic indeed are the glimpses given in these pages of the clouded lives of the babies in the poorest parts of our great city.

But the description of the "Children's Ward" in the big hospital with which these stories begin, if sad enough, has yet its bright side in the thought of the unceasing devotion of the army of men and women who struggle bravely to stem the tide of disease and suffering. The children's wards are "the brightest wards in all the hospital; there are more pictures on the walls, more plants in the windows, and gayer quilts on the beds than in any other part of the building. Fifty little cribs are ranged in rows down the walls, and in nearly every crib a small child, clad in a scarlet jacket, is sitting or lying. Here and there between the tables which occupy the centre of the ward are swinging cradles with red curtains. . . . Altogether, the effect is one of brightness, light, and space."

Perhaps one of the best of the stories from an artistic point of view is the shortest of all, which tells how Euphemia, "the ward cat," brought to the children's ward in the first instance by a confiding student as a temporary patient with a "fractured thigh," finds her new quarters so much to her liking that she is finally abandoned by her lawful possessors and allowed to adopt "the congenial *rôle* of a permanent hospital resident."

"Mrs. Jones" is a pretty little picture of motherly love and self-sacrifice. Poor little Poppet, a small patient, excites herself so much over her mother's visits that the latter heroically agrees not to go near the child whilst harmful results are feared, provided she may come on visiting days and watch her pet from a safe distance behind an intervening screen. "And for ten long weeks did the gaunt, careworn woman keep her word. . . . When convalescence began, and the child was able to play with some toys, an occasional ripple of laughter caused Mrs. Jones to start forward unconsciously, but she always retired hastily to her ambush; and though the tears might sometimes lie wet on her cheeks, she never removed her eyes from the cot where Poppet lay. She

**THE BOOK WORLD FOR WOMEN AND NURSES**—*continued.*

was the first visitor in the ward and the last to leave it when the bell rang out at five o'clock; and she always stood during the whole two hours, and scorned Nurse's offer of a chair with "No, thank'ee Nurse; I could'na bide on it. I sees her best standin'."

The story of the child rescued from a trapeze accident by "Mr. Lolo," the student who was the "children's favourite," and tenderly watched over, cared for, and brought back to a happier life than had ever been his lot before, brings the volume to an end. "Chris was a queer little bit of humanity, with none of the usual childish graces about him. He refused with scorn the toys the nurses offered him, and his sole amusement was to keep a sharp look out on all that went on around him. One day the nurse asked him to watch that none of the semi-convalescent children went to her cupboard, and shortly after Sister Mona came along and took a bandage out of that receptacle. Chris said nothing then, but as soon as Nurse came back he remarked curtly, "Sister's been and nicked somethin' out o' that cupboard o' yourn!" Poor Chris evidently saw nothing strange in the thought that a sister should steal from a nurse!

Space forbids further comment on the other equally attractive stories, some of which are illustrated with full-page drawings; but all are interesting—to hospital workers especially so—and we heartily wish the book all success, and look forward to further glimpses into "the Children's Ward" from the same authors.

---

The fifth edition of Mrs. Brightwen's charming book, "Wild Nature Won by Kindness" (T. Fisher Unwin), is sure to find a ready welcome, not only among members of the Selborne Society. A volume such as this does far more than penalty and prosecution (unavoidable though these may be) in creating a healthy tone about animals in the rising generation. And especially in the treatment of pets, a matter which the omniscient society can rarely touch, Mrs. Brightwen is full of sensible and kindly suggestion. She is catholic in her tastes, writing even of "An Earwig Mother" with cordiality, and her anecdotes extort a measure of respect for the humblest forms of animal life which have come under her observation.

---

"A True Cornish Maid," by G. Norway (Blackie and Son), professes to give a true picture of Cornish life in the last century, but we fear it is in many respects a mere fancy sketch. The character of the heroine, with her keen sense of honour and deep religious spirit, combined with daring courage and resource, is very attractively drawn. But her state of general culture and education are as inconceivable for a fisher-maid of that epoch as the absurd Dolly Varden costume in which she appears in the illustrations. The real condition of the Cornish folk a hundred years ago hardly makes altogether pleasant reading, for it is evident that a large proportion of them were little better than half-naked savages. A people sunk in darkest ignorance and hardened by hundreds of years of brutal custom, do not converse in rounded periods nor get through their skirmishes in kid gloves with feelings of the deepest consideration for each other. There is an odd air of refinement over the whole action which gives the impression that ladies and gentlemen are masquerading as peasants, and that press men and smugglers are excellent fellows making believe to be wicked. Yet the story is told with some spirit, and leaves a conviction that on surer ground, in less wild surroundings, the author could achieve a solid measure of success.

